# Plasma neurofilament light chain, cognition, and MRI measures in community‐dwelling Hispanics

**DOI:** 10.1002/alz70856_101386

**Published:** 2025-12-24

**Authors:** Raul Vintimilla, James Hall, Sid E. O'Bryant

**Affiliations:** ^1^ Institute for Translational Research, University of North Texas Health Science Center, Fort Worth, TX, USA

## Abstract

**Background:**

Plasma neurofilament light chain (NfL), a cytoskeleton protein used as a biomarker of neurodegeneration, has been getting attention in research as a promising biomarker of AD. No large‐scale research of NfL and its association with cognition and MRI biomarkers have been conducted in community‐dwelling Hispanics.

**Method:**

Data from 763 Hispanics, [618 cognitively unimpaired (CU) and 145 with mild cognitive impairment (MCI)] were analyzed. Fasting blood samples were collected and stored at ‐80^0^. NfL was assayed using the ultra‐sensitive SIMOA technology platform HD‐1 (Quanterix.com). Measures of memory (Spanish and English verbal learning test), executive function (Trails B), verbal fluency with (FAS and animals naming), and processing speed (digit symbol substitution) were obtained. Participants underwent a 3T MRI following ADNI 3 protocol. Regional cortical thickness (for calculation of signature meta‐ROI), and hippocampal volume were derived using FreeSurfer software package. White matter hyperintensities (WMH) volume was measured from FLAIR using the Statistical Parametric Mapping Lesion Segmentation Tool. To examine correlations between NfL level and demographic characteristics, Pearson correlation was used. Linear regression models were utilized to examine the associations of log transformed plasma NfL with cognitive (z‐scores) and neuroimaging measures (log transformed). Model 1 was adjusted for age, sex, and education. Model 2 was additionally adjusted for the presence of hypertension, diabetes and dyslipidemia. Neuroimaging models were also adjusted for intracranial volume and scanner differences.

**Result:**

Except for dyslipidemia, NfL level was significantly correlated with all covariates (*p* ≤ 0.05). After adjusting for demographic and vascular covariates, Nfl was significantly associated with delayed memory and procession speed measures (*B* = ‐0.16, *p* =  0.009; B = 0.012, *p* =  0.02 respectively). Higher plasma NfL level was significantly associated with greater meta‐ROI (*B* = ‐0.01, *p* =  < 0.001), and WMH volumes (*B* = 0.02, *p* =  0.01). When separated by CU and MCI groups, findings went away for cognitive measures but hold for neuroimaging measures.

**Conclusion:**

NfL level associations were limited to delayed memory, processing speed, meta‐ROI and WMH. Cognitive status in community‐based samples need to be considered to better understand the relationship of NfL with cognition and brain measures in Hispanics.

